# Dynamic elementary mode modelling of non-steady state flux data

**DOI:** 10.1186/s12918-018-0589-3

**Published:** 2018-06-18

**Authors:** Abel Folch-Fortuny, Bas Teusink, Huub C.J. Hoefsloot, Age K. Smilde, Alberto Ferrer

**Affiliations:** 1 Grupo de Ingeniería Estadística Multivariante, Departamento de Estadística e IO Aplicadas y Calidad, Universitat Politècnica de València, Valencia, Spain; 2 Genetics BioIT DBC Department, DSM Food Specialties, Delft, The Netherlands; 3 Systems Bioinformatics, Centre for Integrative Bioinformatics, Free University of Amsterdam, Amsterdam, The Netherlands; 4 Biosystems Data Analysis, Swammerdam Institute for Life Sciences, University of Amsterdam, Amsterdam, The Netherlands

**Keywords:** Metabolic network, Elementary mode, Dynamic modelling, Principal component analysis, Principal elementary mode analysis, Partial least squares regression discriminant analysis, N-way, Cross validation

## Abstract

**Background:**

A novel framework is proposed to analyse metabolic fluxes in non-steady state conditions, based on the new concept of dynamic elementary mode (dynEM): an elementary mode activated partially depending on the time point of the experiment.

**Results:**

Two methods are introduced here: dynamic elementary mode analysis (dynEMA) and dynamic elementary mode regression discriminant analysis (dynEMR-DA). The former is an extension of the recently proposed principal elementary mode analysis (PEMA) method from steady state to non-steady state scenarios. The latter is a discriminant model that permits to identify which dynEMs behave strongly different depending on the experimental conditions. Two case studies of *Saccharomyces cerevisiae*, with fluxes derived from simulated and real concentration data sets, are presented to highlight the benefits of this dynamic modelling.

**Conclusions:**

This methodology permits to analyse metabolic fluxes at early stages with the aim of i) creating reduced dynamic models of flux data, ii) combining many experiments in a single biologically meaningful model, and iii) identifying the metabolic pathways that drive the organism from one state to another when changing the environmental conditions.

**Electronic supplementary material:**

The online version of this article (10.1186/s12918-018-0589-3) contains supplementary material, which is available to authorized users.

## Background

Data analysis methods are widely used in Systems Biology to interpret different kinds of data. In the field of fluxomics, principal component analysis (PCA) [1] models have been proposed to obtain a set of key pathways in metabolic networks, assuming steady state conditions [2, 3]. Basically, these key pathways are groups of correlated metabolic fluxes measured in different experiments. Multivariate curve resolution (MCR) [4] was afterwards proposed to obtain this set of metabolic pathways, exploiting the ability of MCR to include constraints in the algorithm, driving the model to a more biologically meaningful solution [5].

The drawback of PCA and MCR is that the components do not represent metabolic routes connecting substrates with end-products, but separate groups of concatenated reactions in the network. To enhance the interpretability of PCA and MCR, principal elementary mode analysis (PEMA) [6] was proposed to build a multivariate model using thermodynamically feasible pathways retrieved directly from the network. In the PEMA model, fluxes from different experiments are projected into the most representative set of elementary modes (EMs) from the metabolic network. The EMs are the simplest representations of pathways in the metabolic network. Basically, each EM connects substrates with end-products concatenating reactions.

In non-steady state conditions, the state of the network at a particular time point of the biological process is defined by the concentration of each metabolite in the cell, and metabolites may interact via one or more reactions. Each reaction is represented by an ordinary differential equation (ODE) relating chemical compounds. Since metabolic networks may have hundreds of reactions, it is hard to build kinetic models requiring kinetic parameters. When given the initial concentrations of metabolites and the full kinetic model (including the values for the kinetic parameters), the concentration of the metabolites along time can be simulated to produce a state transition path or trajectory, i.e. the succession of states adopted by the network over time [7]. Methodologies commonly applied when dealing with the aforementioned ODE systems, however using different data sources, are kinetic modelling [8], dynamic flux balance analysis (DFBA) [9], and a recently proposed approach combining time-resolved metabolomics and dynamic FBA (MetDFBA) [10], among others.

Once the kinetic model is built and the data is gathered, either simulated or (partially) measured, a comparison between experimental conditions can be performed to discover which groups of metabolites, reactions or pathways show differences between substrates, environment, etc. For this purpose, partial least squares regression discriminant analysis (PLS-DA) [11] can be used to find metabolites that are strongly related to a response variable (e.g. group of experiments) [12]. The problem with this approach is that no topological information is included in the multivariate model. The identified metabolites can be scattered in the network, not showing clear metabolic routes, as it happened in PCA with steady state data.

The Goeman’s test was proposed in [13] to tackle the lack of topological information in the PLS-DA model. In that case, discrimination between experiments using metabolite concentrations was investigated using the set of pathways retrieved from the Kyoto Encyclopedia of Genes and Genomes (KEGG) database [14–16]. The aim was to find which pathways have a different activation pattern depending on the initial conditions of the experiment at particular time points. This model includes topological information, as metabolites are tested in groups of KEGG pathways, but these pathways sometimes do not connect directly substrates with end products, and the model is not built including all pathways and time points simultaneously.

To solve the aforementioned drawbacks of PLS-DA and the Goeman’s global test, a novel framework is proposed to analyse non-steady state metabolite concentrations, based on an extension of the PEMA model. For this, we introduce the concept of dynamic EMs (dynEMs), i.e. EMs activated partially at each time point of the experiment. The dynEMs are used in a discriminant model to identify which metabolic routes have different activations depending on the initial conditions, i.e. which pathways discriminate between experimental conditions (as for example different substrate concentrations). As opposed to PLS-DA, dynEMR-DA integrates topological information to make the model more interpretable, as the set of candidates are drawn from the elementary mode matrix of the metabolic network; and, as opposed to Goeman’s test, includes all metabolic routes connecting substrates with end-products and all time points of the experiment in the same discriminant model.

The MATLAB code for dynEMR-DA, related functions and example data are freely available in http://www.bdagroup.nl/content/Downloads/software/software.php, with instructions about how to use the method with own data. This way, practitioners are guided through the procedure, from the definition of the inputs, elementary mode matrix and concentration or flux data (either can be used), to the outputs, i.e. coefficients for the dynamic elementary modes to reconstruct the flux data. The *N*-way toolbox [17] and *efmtool* [18] for MATLAB are required to use dynEMR-DA code.

The structure of the article is as follows. In Methods, the metabolic models and data sets of *S. cerevisiae* are presented and the adaptation of the PEMA model from a steady to a non-steady state environment is introduced, describing dynEMA, dynEMR-DA and the validation scheme. In Results, the output of dynEMR-DA is analysed using simulated and real concentration data. Finally, some conclusions are drawn in the last section.

## Methods

### Metabolic networks

**Fig. 1:**
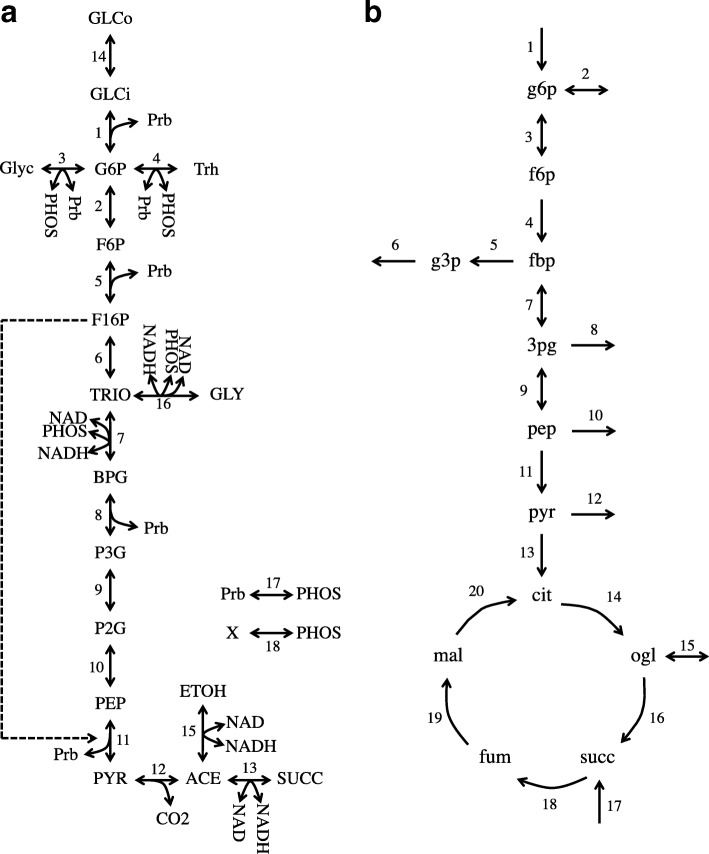
*S. cerevisiae* metabolic models. Model **a**), from [19], is used for the simulated study, and **b**), from [13], for the real case study

Two metabolic models of the well-known baker’s yeast *S. cerevisiae* are used here to build the multivariate discriminant models (see Additional file 1 for a list of reactions). The first one was used in [19] to study the dynamics in glycolysis. The metabolic network (see Fig. 1a) has *M*=23 metabolites and *K*=18 reactions. This metabolic model has 26 elementary modes.

The second model was proposed in [10], and comprises *M*=12 metabolites and *K*=20 reactions, and describes the glycolysis and the tricarboxylic acid (TCA) cycle (see Fig. 1b). This second metabolic model has 13 elementary modes.

Two models are used in this article since the metabolites whose measurements were available in the real case study were not exactly the same as in the simulated model. Also, kinetic parameters were only available for the simulated case study. However, since both models are describing glycolysis in the same organism, the results are comparable.

### Concentration data

The concentration data used in the first model (Fig. 1a) are simulated using COmplex PAthway SImulation (COPASI) software [20]. The initial concentrations of the metabolites match the measurements used in the original paper [19] (see Table 1). In this case, COPASI is used to simulate the concentrations from 0 to 60 s in 20 intervals of 3 s using a deterministic method (LSODA) [21]. The metabolic fluxes and the set of EMs are also obtained directly from COPASI.

**Table 1:** Initial concentrations in the simulated study. Experimental conditions taken from [19]

Metabolite	Initial concentration (mMol/l)
GLCi	0.087
Prb	5
G6P	3.085
F6P	0.75247
Glyc	0
PHOS	10
Trh	0
F16P	0.836
TRIO	0.5177
NAD	0
BPG	0.111
NADH	0.044
P3G	0.825
P2G	0.13771
PEP	0.1404
PYR	0.884031
ACE	0.0474837
CO2	1
SUCC	0
GLCo	110
ETOH	0
GLY	0.15
X	0

The aim in the simulated study consists of discriminating between scenarios using a high *versus* low initial concentration of glucose. 64 experiments are simulated using the data in Table 1, plus 20% noise, that is: *c*=(1+0.2*ε*)*c*_0_, where *c* is the concentration used in the analysis, *c*_0_ is the concentration given by COPASI and *ε* follows a Normal distribution with mean 0 and standard deviation 1. In the first 32 experiments the initial glucose concentration is set to 10mMol/l (plus noise), while in the last 32, this concentration is set to 2.5 mMol/l (also adding noise). These two values are indeed interesting, since they mimic the glucose concentrations used in the real case study (see paragraph below). The other common metabolites between metabolic models have comparable values in both concentration data sets. The set of EMs is obtained in this case using *efmtool* software [18].

In the real case, the concentrations of *S. cerevisiae* along 24 time points were obtained experimentally using liquid chromatography–mass spectrometry (LC-MS) [22, 23] at the Biotechnology Department of Delft University of Technology (The Netherlands), and were used afterwards in [13]. 12 different cultures are used in the present work (see Table 2). Regarding experiments 1 to 8, different initial glucose concentrations in aerobic conditions were used in these cultures: 10 mMol of glucose were used in the first 4 experiments and 2.3-2.5 mMol in experiments 5-8. Also, 4 more cultures, experiments 9 to 12, were performed using similar initial glucose concentrations as in experiments 5-8 but in anaerobic conditions (see Availability of data and materials section for more information on these data).

**Table 2:** Experiments used for the real case study. More details in Availability of data and materials section and in [13, 22, 23]

Experiment number	Aerobic/anaerobic	Conditions
1	Aerobic	10 mM glucose
2	Aerobic	10 mM glucose
3	Aerobic	10 mM glucose
4	Aerobic	10 mM glucose
5	Aerobic	2.5 mM glucose
6	Aerobic	2.5 mM glucose
7	Aerobic	2.3 mM glucose
8	Aerobic	2.3 mM glucose
9	Anaerobic	Glucose deprivation (feed off)
10	Anaerobic	1 mM glucose
11	Anaerobic	3 mM glucose
12	Anaerobic	3 mM glucose + 3 mM acetaldehyde

The aim in the real case study consists of discriminating between i) high and low glucose concentrations (i.e. experiments 1-4 vs 5-8), and ii) aerobic and anaerobic conditions (experiments 5-8 vs 9-12).

### Notation

Scalar values are represented here as italic capital letters (e.g. *N*) and indices will appear as italic lower-case letters (e.g. *j*). Vectors are represented as bold lower-case letters (e.g. **v**). Data matrices are represented as bold capital letters (e.g. **X**). Superindex ^T^ denotes the transpose of a matrix. Observations or individuals within matrices are represented by rows, while variables are represented as columns. 3-dimensional arrays will be denoted as underlined bold capital letters (e.g. **X**). The mathematical operator × is used here to denote the size of the modes of a matrix (e.g. **Y** is a *N*×*M* matrix). No mathematical operator is used for products between scalars, vectors and matrices. Operator ∘ denotes the Hadamard element-wise product between vectors or matrices. Finally, operator ⊗ denotes the Kronecker tensor product between vectors or matrices, that is:

1 $$ \mathbf{X}\otimes \mathbf{Y}=\left[\begin{array}{cc} x_{11} & x_{12} \\ x_{21} & x_{22} \end{array}\right] \otimes \mathbf{Y}=\left[\begin{array}{cc} x_{11}\mathbf{Y} & x_{12}\mathbf{Y} \\ x_{21}\mathbf{Y} & x_{22}\mathbf{Y} \end{array}\right]  $$

Squares and rectangles are used in figure drawings as a representation of matrices.

### Dynamic elementary mode analysis (dynEMA)

Any steady state flux distribution **x**=(*x*_1_,…,*x*_*K*_) can be decomposed as a positive linear combination of a set of *E* EMs [24]:

2 $$ \mathbf{x}=\sum\limits_{e=1}^{E} \lambda_{e}\mathbf{p}_{e}  $$

where *K* is the number of fluxes (matching the number of reactions in the network), $\phantom {\dot {i}\!}\mathbf {p}_{e}=(p_{e_{1}},\ldots,p_{e_{K}})$ is the *e*th EM, *λ*_*e*_ is the positive weighting factor of the *e*th EM, and *E* is the number of EMs needed to reconstruct the flux distribution **x**. The set of *E* EMs is a subset of the complete set of *Z* EMs of the metabolic network.

**Fig. 2:**
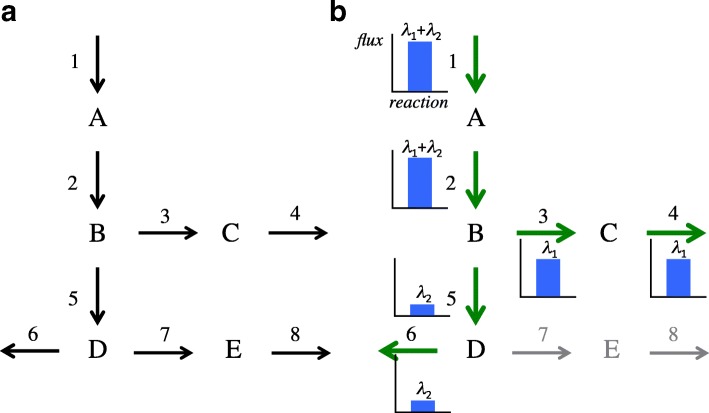
**a** Small metabolic network. **b** Steady state flux distribution. In **b**), the flux carried by each reaction is shown. Reactions 7-8 have no flux

Figure 2a shows an example of this modelling using a small network with *M*=5 metabolites and *K*=8 reactions. There are *Z*=3 EMs in the network: (1,1,1,1,0,0,0,0), (1,1,0,0,1,1,0,0) and (1,1,0,0,1,0,1,1). Let us assume that there is only flux on reactions 1 to 6. A linear combination of the first *E*=2 EMs will reconstruct the flux carried by the reactions in the system in Fig. 2b. In this case, all reactions in each EM are multiplied by the same value. The weighting factors correspond to the flux shown in the graphics beside reactions.

**Fig. 3:**
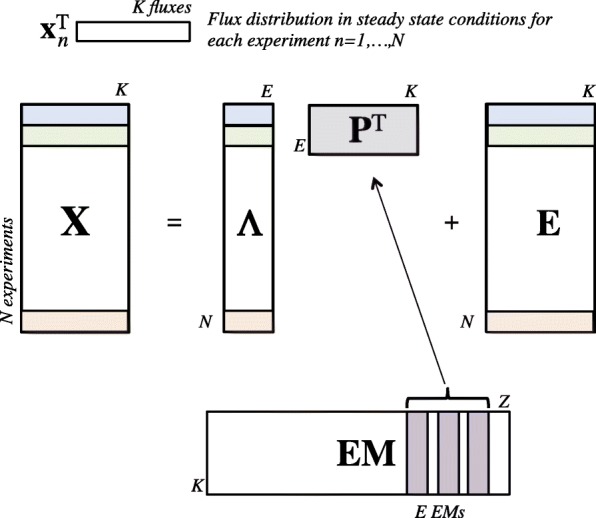
Schematic representation of data matrices in the PEMA model

When *N* flux distributions are considered, coming from different experiments or cultures, a PEMA model can be built:

3 $$ \mathbf{X}=\boldsymbol{\Lambda}\mathbf{P}^{\mathrm{T}}+\mathbf{F}  $$

where **X** is the *N*×*K* flux data matrix, **P** is the *K*×*E* principal elementary mode (PEM) matrix, formed by a subset of *E* EMs; ***Λ*** is the *N*×*E* weighting matrix; and **F** is the *N*×*K* residual matrix. A schematic representation of a PEMA model is shown in Fig. 3.

Non-steady state flux distributions cannot be decomposed as linear combinations of EMs, as in steady state. When the biological system has not reached yet the steady state, the system is not in equilibrium and fluxes can change over time. However, the EMs are indeed the simplest pathways along which the non-steady state fluxes have to flow, but not in a constant fashion. Thus, the EMs must be modified or adapted to fit this dynamical system. These are the so-called dynamic elementary modes (dynEMs).

To adapt an EM, there is not only a single coefficient multiplying the EM (***Λ*** values in PEMA):

4 $$ \lambda_{e}\mathbf{p}_{e}=(\lambda_{e} p_{e_{1}},...,\lambda_{e} p_{e_{K}})  $$

but a different coefficient multiplying each reaction activated by the EM:

5 $$ \boldsymbol{\alpha}_{e_{j}}\circ\mathbf{p}_{e}=(\alpha_{e_{j,1}} p_{e_{1}},\ldots\alpha_{e_{j,K}} p_{e_{K}})  $$

where $\boldsymbol {\alpha }_{e_{j}}$ includes the coefficients that adapt reactions 1 to *K* in the selected *e*th dynamic EM to reproduce the metabolic fluxes at time point *j*, and ∘ is the Hadamard element-wise product of matrices.

Thus, a single non-steady state flux distribution **x** at time point *j* can be decomposed as:

6 $$ \mathbf{x}_{j}=\sum\limits_{e=1}^{E} \boldsymbol{\alpha}_{e_{j}} \circ \mathbf{p}_{e}   $$

**Fig. 4:**
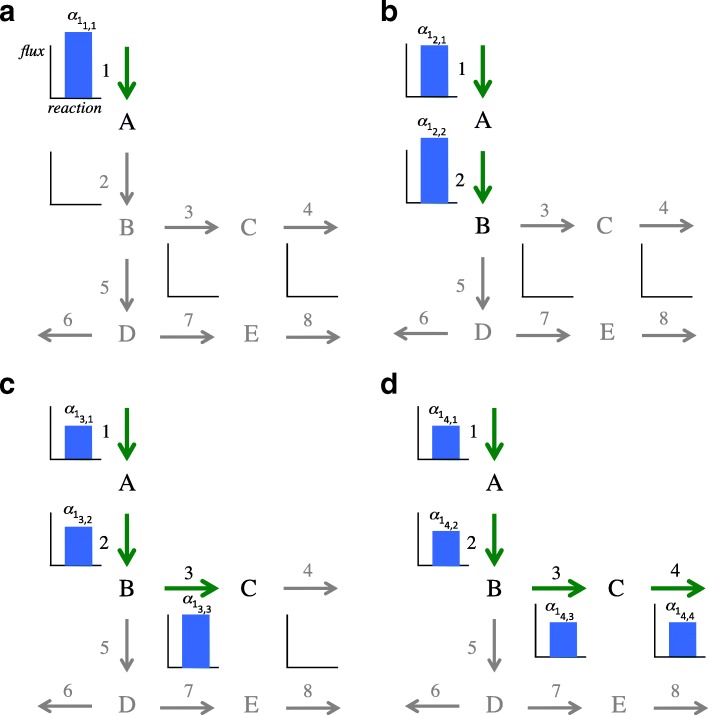
Small metabolic network with non-steady state fluxes from time point 1 to 4 (**a**) to **d**), respectively). Graphics show the flux carried by each reaction, which changes depending on the time point. The first subindex of the weighting factor $\alpha _{e_{j,k}}$ indicates the EM *E*=1. The other two subindices indicate time point *j*=1,..,4 and reaction *k*=1,..,8

Consider now a set of non-steady state flux distributions, which can be obtained from a single experiment measuring the concentration of the metabolites at *J* consecutive time points. Figure 4 shows an example of this scenario using the previous small network. Let us assume that there are fluxes only in reactions 1 to 4. In this case, only *E*=1 EM is needed. However, at each time point (*j*=1,…,4) the flux at each reaction (*k*=1,…,8) is different. High values are registered at the beginning of the experiment in the first reaction (Fig. 4a). Afterwards, the flux reaches all metabolites in the EM (Fig. 4b-c). Finally, the experiment reaches the steady state at the last time point (Fig. 4d), and all fluxes in the reactions are similar.

**Fig. 5:**
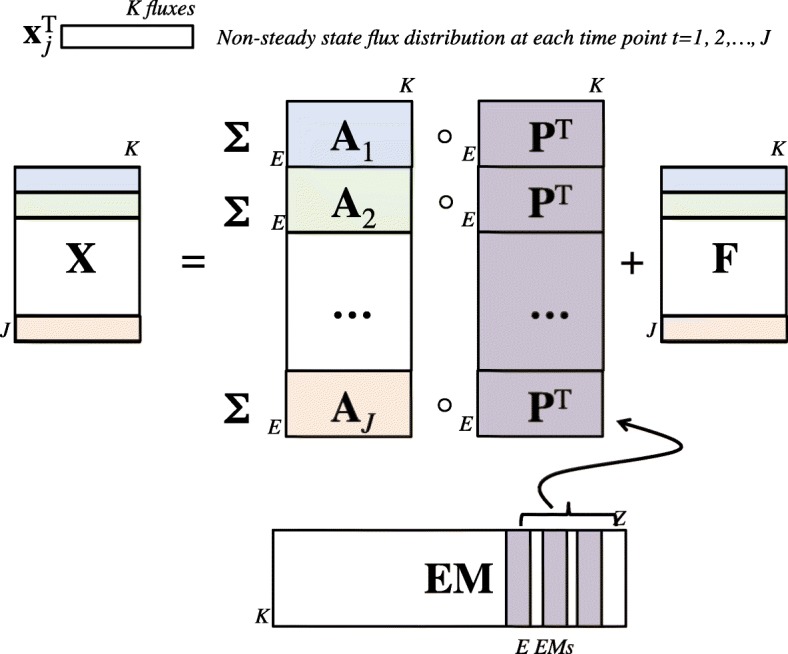
Schematic representation of data matrices in the dynEMA model

Considering non-steady state flux distributions along *J* time points, the set of active dynEMs can be obtained, in a PEMA/PCA-like fashion, from the new dynamic elementary mode analysis (dynEMA) model:

7 $$ \mathbf{X}=(\mathbf{I}_{J}\otimes\mathbf{1}^{\mathrm{T}}_{E}) \lbrack \mathbf{A}\circ(\mathbf{1}_{J}\otimes \mathbf{P}^{\mathrm{T}})\rbrack + \mathbf{F}   $$

where **A** is the *EJ*×*K* coefficients matrix, **I**_*J*_ is the *J*×*J* identify matrix, **P** is the *K*×*E* principal elementary mode (PEM) matrix, **1**_*E*_ and **1**_*J*_ represent column vectors of *E* and *J* ones respectively, **F** is the *J*×*K* residual matrix (containing the fluxes not explained by the set of dynamic elementary modes) and ⊗ is the Kronecker matrix product. In this case, **X** is a *J*×*K* data matrix representing the non-steady state fluxes from a single experiment along *J* time points; while in the PEMA model, **X** is a *N*×*K* matrix representing the steady state fluxes of *N* different experiments. Figure 5 shows a representation of dynEMA model.

The coefficients matrix **A** in the previous equation is, in fact, a *E*×*K*×*J* 3-way matrix unfolded reaction-wise, and each entry in the matrix $\alpha _{e_{jk}}$ represents the coefficient multiplying reaction *k* of EM *e* to reconstruct the flux at time point *j*. Using this modelling it is possible to study the time evolution of a dynEM, i.e. how the dynEM is adapted or dynamically used along all measured time points for a given experimental condition.

This system of equations is solved similarly to PEMA. The candidates for first dynEM are selected from the complete *K*×*Z***EM** matrix in a step-wise fashion. After selecting an EM, the coefficients multiplying it (thus creating the dynEM) are obtained solving Eq. 7 using non-negative least squares. Once all EMs are evaluated, the dynEM explaining most variance in data (as in PEMA) is classified as the first dynEM (1st column of PEM matrix **P**). Afterwards, this first dynEM is set, and the search for the second one starts, recalculating the coefficients in matrix **A** for both the first and the second dynEMs at each evaluation. In this way, the dynEMA model is built in a greedy way, explaining as much variance as possible at each step.

Regarding the number of dynEM extracted, this depends on the aim of the analysis, as explained in [6] with the PEMA model. For example, when the aim is to identify the main dynamic behaviour, one dynEM is enough. If the aim is to identify the main dynEM utilizing one particular section of the network, the model needs as many dynEMs as required to represent those reactions. Alternatively, one can extract as many dynEMs needed to reach certain percentage of explained variance (e.g. 95%).

The dynEMA model is useful to identify the dynEMs active in an experiment and how each dynEM is used in the culture at different time points of the experiment.

### Dynamic elementary mode regression discriminant analysis (dynEMR-DA)

When the aim is to establish differences between environmental or experimental conditions, e.g. presence/absence of a compound or case/control studies, a discriminant model is needed. For this, dynamic elementary mode regression discriminant analysis (dynEMR-DA) is proposed here. This model focuses on finding which are the dynEMs with a strongly different time evolution or performance between conditions. In essence, dynEMR-DA is a two-step procedure. First, it projects the flux data into the space defined by each single dynEM. Then, fits a NPLS-DA [25] model with discriminant purposes.

**Fig. 6:**
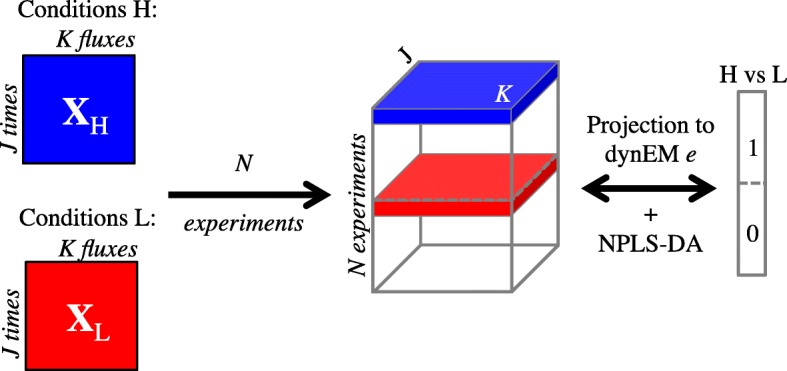
dynEMR-DA procedure. **X**_H_ and **X**_L_ denote the flux data matrices of two different experimental conditions

To build a dynEMR-DA model, the set of different experiments are combined in a single $\underline {\mathbf {X}}$ 3-way matrix (see Fig. 6). In **X** we consider *N* experiments, measuring *K* fluxes along *J* time points. Therefore, it is mandatory to have the same time points in all experiments.

The algorithm of dynEMR-DA has the following steps:

1. For each EM in the metabolic network (candidate to dynEM):
  - Unfold reaction-wise the *N*×*K*×*J***X** matrix in Fig. 6 in a two-way *JN*×*K* matrix **X**.
  - Calculate the coefficients matrix **A** using the dynEMA model:
    8 $$ \mathbf{X}=\left(\mathbf{I}_{JN}\otimes\mathbf{1}^{\mathrm{T}}_{E}\right) \left\lbrack \mathbf{A}\circ\left(\mathbf{1}_{JN}\otimes \mathbf{p}^{\mathrm{T}}\right)\right\rbrack + \mathbf{F}   $$
    where **p** denotes the candidate EM from step 1.
  - Reconstruct the flux data $\hat {\mathbf {X}}$ using the dynEMA model:
    9 $$ \hat{\mathbf{X}}=\left(\mathbf{I}_{JN}\otimes\mathbf{1}^{\mathrm{T}}_{E}\right) \left\lbrack \mathbf{A}\circ\left(\mathbf{1}_{JN}\otimes \mathbf{p}^{\mathrm{T}}\right)\right\rbrack   $$
  - Fold the reconstructed data to build again a three-way data structure $\underline {\hat {\mathbf {X}}}$
  - Fit an NPLS-DA model between the reconstructed data and the **y** data, where **y** denotes the class of experiments (having 1s and 0s).
2. The dynEM whose NPLS-DA model explains most variance in **y** is classified as the first dynEM.
3. Check the predictions of NPLS-DA model. If the current model discriminates perfectly, stop. If not, set the first dynEM and repeat steps 1-3 to extract the second dynEM following the dynEMR-DA procedure.

NPLS-DA was proposed for studying *N*-dimensional data structures with discriminant purposes. NPLS is the natural extension of PLS to *N*-way structures, which tries to maximize the covariance between the $\underline {\mathbf {X}}$ and **Y** data arrays. **Y** is denoted as **y** when one variable is predicted. NPLS-DA models in this paper have been computed using the *N*-way toolbox for MATLAB [17].

The dynEMR-DA algorithm can select many dynEMs until attaining a perfect discrimination. However, in practice, individual dynEMs are able to discriminate between two experimental conditions, so there is no need of considering two dynEMs simultaneously active to obtain a discriminant model. Moreover, some dynEMs are discriminating between initial conditions, but some of their reactions are not used at any time point of the experiment (so the flux does not flow through the metabolic pathway from the beginning to the end). These dynEMs do not represent actual metabolic pathways, so they should be removed when they are selected.

### Triple cross-validation (3CV)

Proper validation of multivariate models is a subtle issue in Systems Biology. When enough data are available, single cross-validation procedures may lead to too optimistic models, especially when the aim is discrimination between classes. As commented in [26], when discriminant models, such as PLS-DA, are used on datasets with much more variables than samples, the models cannot be built as accurately as when there are more samples than variables. Then, the high number of variables can lead to chance discriminations, i.e. models that give good results because a variable had by chance lower values in all samples from one group. To avoid this sometimes spurious results, double cross validation (2CV) was proposed [26]. Using this procedure, a subset of the original data is used to model fitting, another subset to decide the complexity of the model (e.g. number of components of a multivariate model), and finally, a third subset is used for validation. This kind of models are especially useful for (N)PLS-DA model validation [26, 27].

**Fig. 7:**
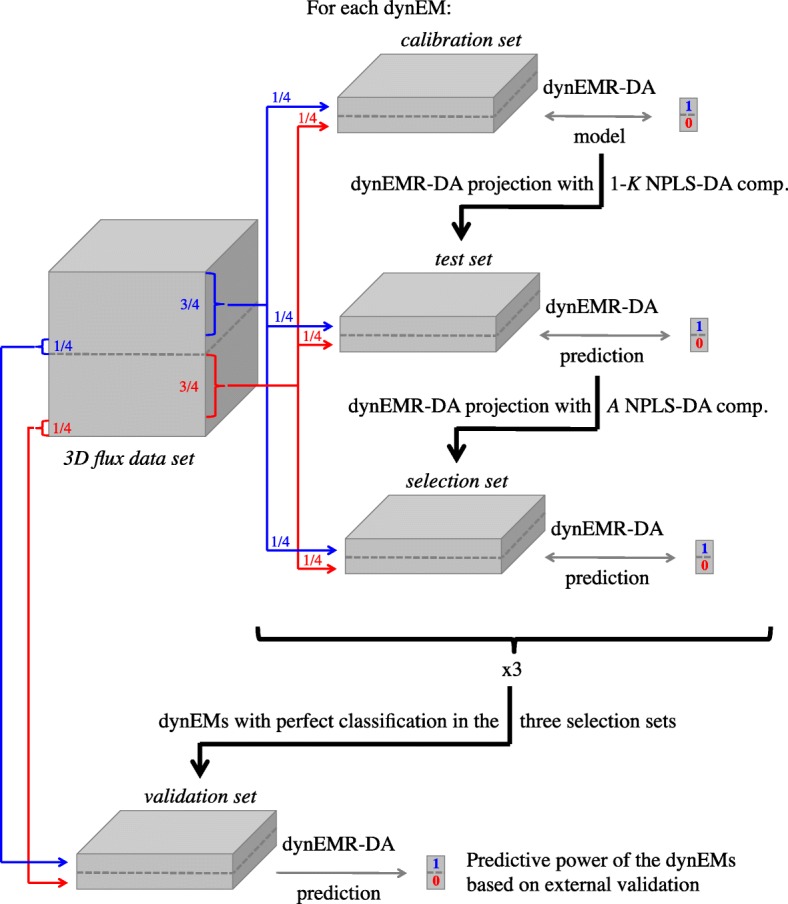
3CV procedure. 75% of the samples from both classes (red and blue) are used in the calibration, projection and test sets (25% in each). The remaining 25% of samples are used in validation set

In this work, though, we need an extra round of validation. dynEMR-DA models involve the projection, as first step, of the flux data into the space defined by each single dynEM. Afterwards, an NPLS-DA model is fitted, determining at the end which dynEMs are discriminating between groups. Therefore, we propose here a triple cross validation (3CV) scheme (see Fig. 7). This procedure consists of the following steps:

1. Divide the data set in four groups: calibration, test, selection, and validation. The latter is left out of the analysis until the final external validation.
2. Fit a dynEMR-DA model using the calibration set, using a maximum of *K* components (as many as fluxes).
3. Project the test set, first to the corresponding dynEM, and then to each of the *K* NPLS-DA calibration models. At this point, the minimum number of components, *A*, needed to classify each experiment in its corresponding class, is selected.
4. Project the selection set into the previous dynEMR-DA model with *A* NPLS-DA components and evaluate the predictive power of each dynEM.
5. Steps 2-4 are repeated three times, changing the roles of the subsets. That is, the models are built using, in steps 2 to 4 respectively: calibration-test-selection, test-selection-calibration and selection-calibration-test sets.
6. The dynEMs with perfect classification rates using the selection set in the three rounds are used finally for validation, so the discrimination power of each dynEM is evaluated with completely external data. This prediction is performed substituting the selection group by these validation samples in the three models previously fitted.

A 2CV strategy is used for the NPLS-DA section of the dynEMR-DA models, but an extra validation round is needed to assess the performance of the selected dynEMs in terms of discrimination. Therefore, the 3CV procedure is built basically replacing the validation step, in the original 2CV, by the selection step, and performing the external validation in the last step.

## Results

### Simulated flux data

The metabolic model of *S. cerevisiae* in Fig. 1a is used in this section to assess the performance of dynEMR-DA on simulated data. 64 experiments are simulated using COPASI, with the initial concentrations described in Methods (see Table 1). Thus, 32 experiments have a high initial concentration of glucose and 32 a low concentration. The fluxes derived from the concentration data, and also the set of EMs of the metabolic model, are also obtained using COPASI.

To validate the discriminant models, the 3CV scheme is used here, using the *N*-way Toolbox for MATLAB [17] to fit the NPLS-DA models. 8 experiments of each class selected at random (16 in total) are used for calibration. 16 more experiments are used to select the number of NPLS-DA components. And 16 more are used as selection samples. As described in Fig. 7, the first 3 subsets are used as calibration, test and selection sets, and then the roles change, i.e. test-selection-calibration and selection-calibration-test (steps 2-4 described in 3CV). Finally, 16 additional experiments are used as validation set.

When applying the dynEMR-DA procedure described in the previous section, only one dynEM (from the whole set of 26 EMs) is able to discriminate perfectly between both experimental conditions: dynEM 8. Finally, the remaining 16 cultures are used for the final validation of this dynEM (see Fig. 7). Again, all experiments are correctly classified in the dynEMR-DA model.

**Fig. 8:**
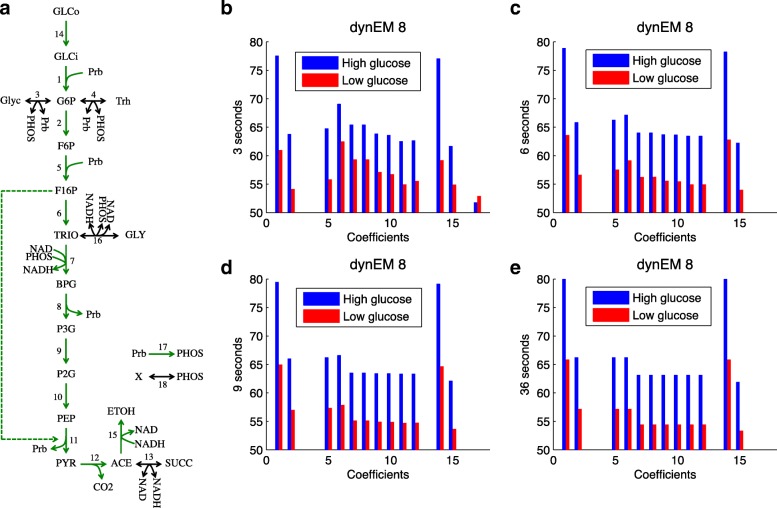
Simulated study. **a** dynEM_8_ depicted on the metabolic model. **b**-**e** dynEM_8_ coefficients at 3, 6, 9 and 36 s (first 3 times points and when the fluxes reach the steady state). Blue (red) lines show the mean of the coefficients for the high (low) glucose experiments

Figure 8a shows dynEM_8_. This mode covers the whole glycolytic pathway, starting from glucose (GLCo), producing all the intermediate products until reaching pyruvate (PYR), acetate (ACE) and finally ethanol (ETOH). The coefficients multiplying the EM are visualized in Fig. 8b-e. The first three time points (3, 6, and 9 s) reveal changes in the coefficients. Afterwards, changes are small. At 36 s, the system reaches the steady state, when fluxes do not change any more.

The differences between both experimental conditions can be seen in Fig. 8b-e (blue *versus* red bars). The usage of all reactions in the dynEM, i.e. the coefficients in **A** matrix, are higher in the high glucose concentration experiments than in the low glucose. This implies that these scenarios take advantage of the higher amount of glucose to carry more flux through the glycolysis until reaching ethanol.

It is worth mentioning that the system is close to steady state from the first time point. However, we used this set up to have a simulated case as close as possible to the real case, in order to find out i) whether there are differences between the initial concentrations of glucose, and ii) if the discriminant dynEM resembles the real case one(s) (see next section).

### Real flux data

#### High vs low initial glucose concentrations

To assess the performance of dynEMR-DA in a real case study, a set of cultures of *S. cerevisiae* are used to discriminate between experiments using a high or a low initial glucose concentration. Unfortunately, the number of available cultures is low for this case study (4 in each class), so no 3CV, neither 2CV, is possible. Therefore, single CV is applied here: 3+3 experiments are used for dynEMR-DA model building and selection of NPLS-DA components, and the remaining 1+1 experiments are used for validation. This procedure is repeated 4 times, leaving out a couple of cultures each time.

The dynEMR-DA model has to be built using fluxes, not concentrations. Therefore, we computed the fluxes based on the changes in the concentrations between two consecutive time points solving an optimization problem (similarly as in [10]). Specifically, the objective function in this formulation makes the fluxes smooth along time (penalizing the sum of the differences between fluxes in consecutive time points) and small (penalizing the sum of squared fluxes), and the constraints force them to fulfil the stoichiometric equations.

In the actual data set, *M*=12 metabolites are measured in 24 time points within 2 min (1 measurement every 3 s). The metabolic network (see Fig. 1b) has *K*=20 reactions. Thus, the optimization problem to solve is:

10 $$ \left\{ \begin{array}{l} \min_{x_{jk}} {\sum\nolimits}_{j=1}^{22} {\sum\nolimits}_{k=1}^{20} (x_{j+1,k}-x_{j,k})^{2} + {\sum\nolimits}_{j=1}^{23} {\sum\nolimits}_{k=1}^{20} x_{j,k}^{2}\\ s.t. \quad \mathbf{S}\mathbf{X}^{\mathrm{T}}=\frac{d\mathbf{C}^{\mathrm{T}}}{dj}\\ \qquad \; \, \mathbf{X}\geq\mathbf{0}\\ \qquad \; \, \mathbf{X}_{0} \: \mathrm{initial \: solution}\\ \end{array} \right.   $$

where **X**={*x*_*jk*_} is the 23×20 (time points × reactions) flux data matrix. The quadratic optimization problem needs an initial guess on **X**, i.e. **X**_0_. This guess is obtained solving $\mathbf {S}\mathbf {X}_{0}^{\mathrm {T}}=\frac {d\mathbf {C}^{\mathrm {T}}}{dj}$ using non-negative least squares. Indices *k* and *j* denote flux number and time point, respectively, **S** denotes the 12×20 stoichiometric matrix (metabolites × reactions), and **C** is the 24×12 concentration matrix (time points × metabolites). It is worth noting that, since fluxes are computed based on the differences between concentrations at consecutive time points, there is one time point less in the flux data matrix (*J*=23) than in the concentration data (24).

The objective function used in the optimization problem resembles the MOMA function (minimize the squared difference of the reaction rates with steady state) used in [10], with the difference that we minimize the flux differences between consecutive time points.

**Fig. 9:**
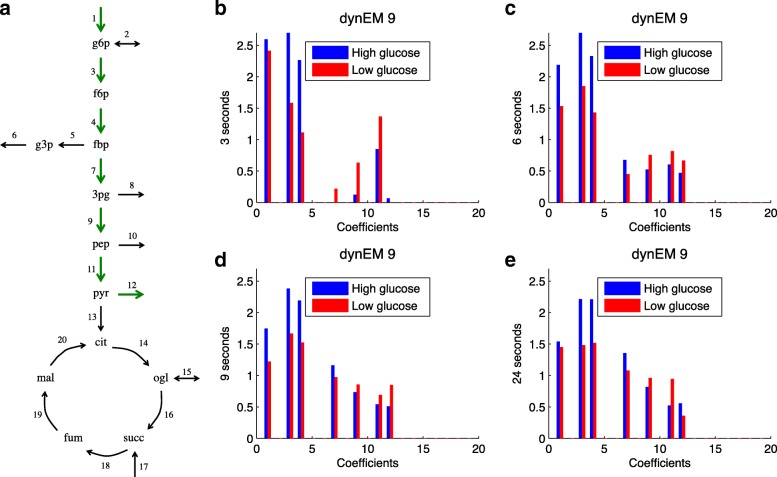
Real case study. **a** dynEM_9_ depicted on the metabolic model. **b**-**e** dynEM_9_ coefficients at 3, 6, 9 and 24 s (when system is close to steady state). Blue (red) lines show the coefficients for the high (low) glucose experiments

In this case, only dynEM_9_ (from the set of 20 EMs) is able to discriminate the left out experiments. This dynEM can be visualised, jointly with the coefficients in matrix **A**, in Fig. 9. The differences between high and low glucose are also clear in this example. The usage of this dynEM is stronger in scenarios with a high initial glucose concentration than with a low concentration.

The results in this example follows the scheme described in Fig. 4. In both experiments (high and low), the fluxes are higher in the first steps of glycolysis (3, 6, and 9 s) and lower at the end. As time goes by, fluxes in the last part of the glycolysis increase. This shows that the flux data cannot be modelled in the same way at the first time points as when the culture reaches the steady state, therefore it necessitates to use of dynEMs to model non-steady state flux data, instead of applying a PEMA-based approach.

It is worth noting the similarity between the dynEM identified here and dynEM_8_ of the simulated case study. Both dynEMs are describing the same phenomena, the glycolysis until reaching pyruvate. They are not exactly the same because the metabolic models are different: acetate and ethanol were not measured in experimental conditions. However, when comparing the simulated and the actual data, the dynEM discriminating between experimental conditions is basically the same one.

Finally, it is difficult to assess when the system reaches the steady state in the real case study. In the simulated case, steady state was reached clearly at 36 s (since fluxes did not change anymore). In the real case, after 24 s (see Fig. 9) fluxes do not change significantly. However, since measurement error is present in the real case, it is difficult to asses whether the steady state was reached at 24 s or afterwards.

#### Aerobic vs anaerobic conditions

For the second real case study, four cultures performed in aerobic conditions *versus* four more in anaerobic conditions are compared. As in the previous example, fluxes are calculated from the real concentration data using the optimization framework (see Equation 10); also, a single cross validation procedure is applied here.

**Fig. 10:**
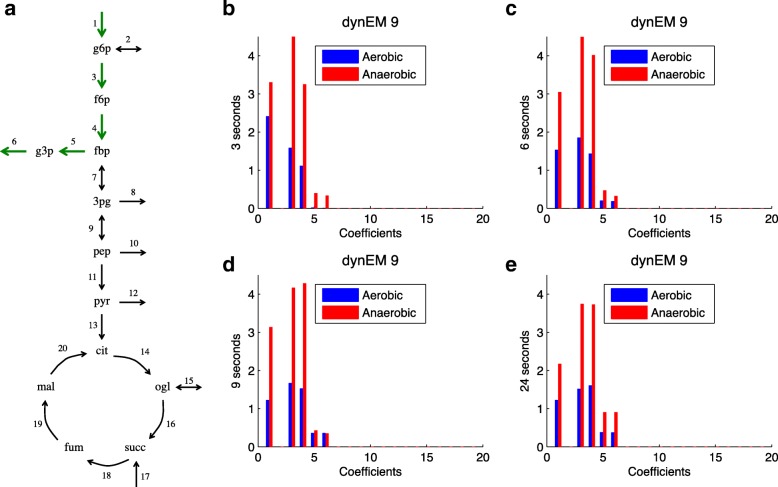
Real case study. **a** dynEM_8_ depicted on the metabolic model. **b**-**e** dynEM_8_ coefficients at 3, 6, 9 and 24 s (when reaching steady state). Blue (red) lines show the coefficients for aerobic (anaerobic) experiments

In this case study, dynEM_8_ is able to discriminate between both experimental conditions. The dynEM and the coefficients at 3, 6, 9 and 24 s (when system seems to reach steady state) can be visualized in Fig. 10. Again, the differences between both classes can be seen in the plots; the anaerobic experiments having higher coefficients. This behaviour has been outlined also in the literature [28–31]. To satisfy the redox balances, the flux is deviated from glycolysis to the production of glycerol (in our case, after reaction 4, flux is going through reactions 5 and 6). Glycerol is produced by reduction of the glycolytic intermediate dihydroxyacetone phosphate to glycerol 3-phosphate (g3p) followed by a dephosphorylation of g3p to glycerol. Despite glycerol does not appear explicitly in the network, because this metabolite was not measured in all original experiments, it is likely that the flux flowing through g3p produce glycerol at the end, as suggested in the literature.

### Comparison to other state-of-the-art techniques

#### NPLS-DA

As in [6], it is worth to compare the approach of an elementary-mode based projection model to a classical projection method, which in this case, is NPLS-DA. To perform this comparison, the real case studies presented in the two previous subsections have been modelled using NPLS-DA algorithm.

**Fig. 11:**
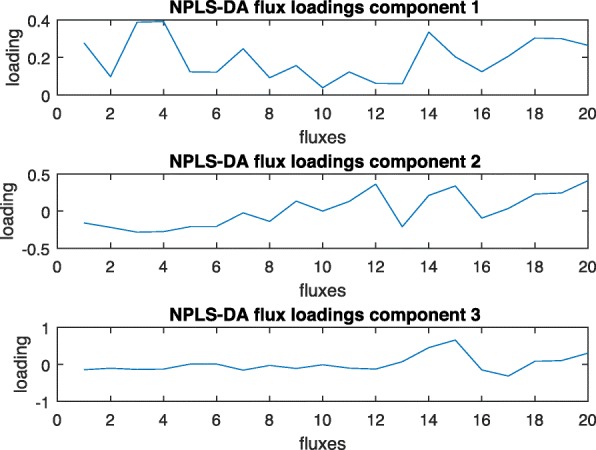
NPLS-DA loading plots for the fluxes (high versus low intial glucose data)

Figure 11 shows the loadings of the fluxes using the high versus low initial glucose data. The model in this case has 3 components, explaining 92 and 95% of variance in flux and discriminant variables, respectively. This number of components corresponds to the most parsimonious model needed to correctly classify all experiments. Firstly, it is difficult to extract from the loading plots which fluxes are the most important for discrimination, as no clear threshold can be drawn in the plot. Secondly, even varying this hypothetical threshold, the significant fluxes (those with high absolute loading coefficient) represent disconnected reactions through the network and do not correspond to physical pathways, since no topological information is included in the model. The NPLS-DA loadings are the elementary modes in dynEMR-DA, therefore interpretation is more straightforward, as they represent real pathways.

**Fig. 12:**
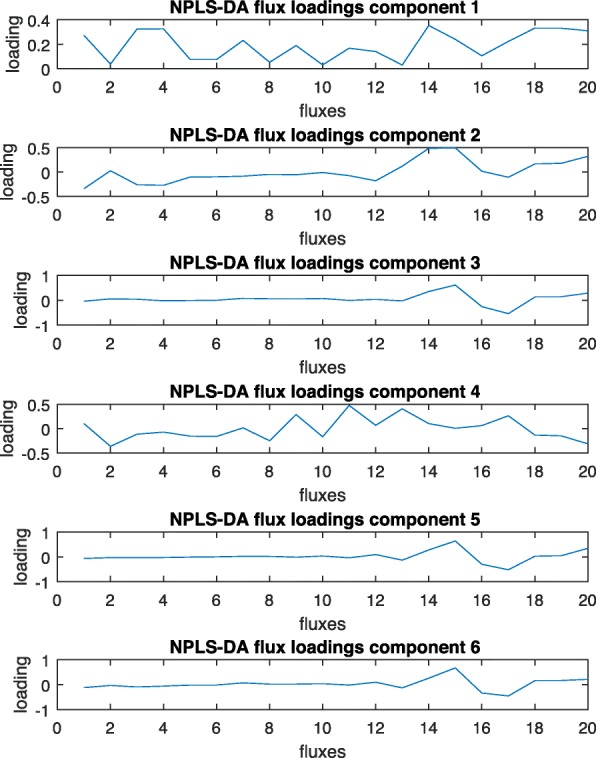
NPLS-DA loading plots for the fluxes (aerobic versus anaerobic data)

Figure 12 shows the results for the aerobic versus anaerobic case study. Here, 6 components are needed, explaining 98 and 99% of variance in flux and discriminant variables, respectively. As in the high versus low initial glucose example, loading plots are very difficult to interpret.

The computation time with these case studies is 17 s (dynEMR-DA model) versus 0.5 s (NPLS-DA model). In the dynEMR-DA algorithm, as many NPLS-DA models as EMs (in this model, 13) are fitted to find the most discriminant one, therefore it is clear that one single NPLS-DA model will be faster than dynEMR-DA. However, the time needed to interpret the output of NPLS-DA is longer than the pathway-oriented result that dynEMR-DA provides.

dynEMR-DA, as opposed to NPLS-DA, can be strongly affected by the size of the EMs matrix. When having several hundreds of EMs, a pre-selection of EMs can be performed to speed up the analysis. One strategy would be to study the reactions that are active in all EMs and include only those EMs with different active reactions (i.e. coefficient different from zero). For example, if many elementary modes use the same reactions with the same directionality for the reversible ones, only one EM can be included in the set of EMs to test. Another possibility would be to use the set of extreme pathways of the network instead of the EMs [24].

#### Goeman’s global test

The Goeman’s global test was applied in [13] to find which KEGG pathways show differences between experimental conditions. The output in that case was a *p*-value indicating which pathways were different depending on the groups at discrete time points. Their results showed that glycolysis and TCA cycle were significant but not for all time points when comparing high versus low initial glucose. For the aerobic versus anaerobic case, both the glycolysis and TCA were significant for all time points.

This approach is not directly comparable to dynEMR-DA, as all pathways are tested simultaneously in dynEMR-DA, instead of individual pathway testing. No EM containing TCA was significant here, which can be also due to i) all time points are used simultaneously in dynEMR-DA, instead of discrete time point analysis (4 time points in [13]), and ii) the dynEMs containing TCA might not show differences between experimental conditions in the non-TCA section of the dynEM.

Finally, authors stated in the Goeman’s test article [13] that a dynamic model would be more suitable for this type of data, which is what was pursued here.

## Discussion and conclusions

The approach for dynamic elementary mode modelling proposed here permits decomposing non-steady state flux distributions into a set of active dynEMs. This way, dynEMA can be used to study the active dynEMs in an experiment, or a set of experiments, extending the PEMA model to a dynamic environment. For discrimination purposes, the main interest in this article, dynEMR-DA allows identifying which dynEMs have different patterns of activation depending on the culture initial conditions.

Actual and simulated concentration data of *S. cerevisiae* have been used here to evaluate dynEMR-DA. When changing the amount of glucose present in the experiment in both data sets, dynEMR-DA is able to identify that the dynEM flowing through the glycolytic pathway from glucose to pyruvate is discriminating between high and low initial glucose concentration experiments. Even considering two different metabolic models, for data availability reasons, the results of dynEMR-DA seem coherent between case studies. When analysing data from aerobic *versus* anaerobic conditions, dynEMR-DA indicates that the most discriminant dynEM drives the initial glucose concentration to the glycerol production. Previously published research confirms the results obtained using this new methodology.

The framework presented here will serve to create reduced dynamic models of flux data while preserving biological and thermodynamical meaning, as a tool to analyse non-steady state flux distributions in many experiments and to identify the hidden metabolic patterns that drive the organism from one state to another when changing the environmental conditions. dynEMA and dynEMR-DA have potential applications in bioprocess engineering to understand the small changes in cell metabolism at early stages of cultures.

## Additional file

Additional file 1 An additional file is provided with the detailed metabolic models. (PDF 105 kb)

## Abbreviations

2CV: double cross-validation
3CV: triple cross-validation
COPASI: complex pathway simulation
CV: cross-validation
DFBA: dynamic flux balance analysis
dynEM(s): dynamic elementary mode(s)
dynEMA: dynamic elementary mode analysis
dynEMR-DA: dynamic elementary mode regression discriminant analysis
EM(s): elementary mode(s)
FBA: flux balance analysis
KEGG: Kyoto Encyclopaedia of Genes and Genomes
LC-MS: liquid chromatography–mass spectrometry
MCR: multivariate curve resolution
MetDFBA: time-resolved metabolomics and dynamic flux balance analysis
NPLS: N-way partial least squares regression
NPLS-DA: N-way partial least squares regression discriminant analysis
ODE: ordinary differential equation
PCA: principal component analysis
PEM(s): principal elementary mode(s)
PEMA: principal elementary mode analysis
PLS: partial least squares regression
PLS-DA: partial least squares regression discriminant analysis
