# Supplementary material for: Dynamic elementary mode modelling of non-steady state flux data
Source: BMC Syst Biol. 2018 Jun 18;12:71. doi: 10.1186/s12918-018-0589-3 (PMC6006576; doi:10.1186/s12918-018-0589-3)
Supplement: Supplementary file 1 — An additional file is provided with the detailed metabolic models. (PDF 105 kb) [file 12918_2018_589_MOESM1_ESM.pdf]

Electronic Supplementary Information for: Dynamic elementary mode modelling of non-steady state flux data

Abel Folch-Fortuny,*[1,2] Bas Teusink,[3] Henk A.L. Kiers,[4] Huub C.J. Hoefsloot,[5] Age K. Smilde,[5] and Alberto Ferrer[1]

1 Metabolic models.

1.1 Simulated case study

Metabolite abbreviations

**Abbreviation**	**Metabolite**
GLCo	Glucose
GLCi	Glucose (intracelullar)
Prb	Energy status
G6P	Glucose-6-phosphate
F6P	Fructose 1,6-phosphate
Glyc	Glycogen
PHOS	Phosphate

[1]Departamento de Estadística e IO Aplicadas y Calidad, Universitat Politècnica de València, Valencia, Spain.

[2]*Genetics BioIT DBC Department, DSM Food Specialties, Delft, The Netherlands.*

[3]*Systems Bioinformatics, Centre for Integrative Bioinformatics, Free University of Amsterdam, Amsterdam, The Netherlands*

[4]*Heymans Institute for Psychology, University of Groningen, Groningen, The Netherlands.*

[5]*Biosystems Data Analysis, Swammerdam Institute for Life Sciences, University of Amsterdam, Amsterdam, The Netherlands.*

*E-mail: abelfolch@gmail.com

Trh	Trehalose
F16P	Fructose-6-biphosphate
TRIO	Triose-phosphates
NAD	Nicotinamide adenine dinucleotide
BPG	Bisphosphoglycerate
NADH	Nicotinamide adenine dinucleotide phosphate
P3G	3-Phosphoglycerate
P2G	2-Phosphoglycerate
PEP	Phosphoenolpyruvate
PYR	Pyruvate
ACE	Acetate
CO2	Carbon dioxide
SUCC	Succinate
ETOH	Ethanol
X	Polyphosphates

List of reactions

GLCi + Prb $\leftrightarrow$ G6P
G6P $\leftrightarrow$ F6P
G6P + Prb $\leftrightarrow$ Glyc + 2 PHOS
Prb + 2 G6P $\leftrightarrow$ Trh + 3 PHOS
F6P + Prb $\leftrightarrow$ F16P
F16P $\leftrightarrow$ 2 TRIO
PHOS + TRIO + NAD $\leftrightarrow$ BPG + NADH
BPG $\leftrightarrow$ P3G + Prb
P3G $\leftrightarrow$ P2G
P2G $\leftrightarrow$ PEP
PEP $\leftrightarrow$ Prb + PYR; F16P
PYR $\leftrightarrow$ ACE + CO2
2 ACE + 3 NAD $\leftrightarrow$ SUCC + 3 NADH
GLCo $\leftrightarrow$ GLCi
ACE + NADH $\leftrightarrow$ ETOH + NAD
NADH + TRIO $\leftrightarrow$ PHOS + GLY + NAD
Prb $\leftrightarrow$ PHOS
X $\leftrightarrow$ PHOS

1.2 Real case study

Metabolite abbreviations

**Abbreviation**	**Metabolite**
g6p	Glucose-6-phosphate
f6p	Fructose-6-phosphate
fbp	Fructose 1,6-biphosphate
g3p	Glyceraldehydes-3-phosphate
3pg	3-Phosphoglycerate
pep	Phosphoenolpyruvate
pyr	Pyruvate
cit	Citric acid
ogl	Oxoaglutarate
succ	Succinate
fum	Fumarate
mal	Malate

List of reactions

g6p $\rightarrow$
g6p $\leftrightarrow$
g6p $\leftrightarrow$ f6p
f6p $\rightarrow$ fbp
fbp $\rightarrow$ g3p
g3p $\rightarrow$
fbp $\leftrightarrow$ 3pg
3pg $\rightarrow$
3pg $\leftrightarrow$ pep
pep $\rightarrow$
pep $\rightarrow$ pyr
pyr $\rightarrow$
pyr $\rightarrow$ cit
cit $\rightarrow$ ogl
ogl $\leftrightarrow$
ogl $\rightarrow$ succ
succ $\rightarrow$
succ $\rightarrow$ fum

fum $\rightarrow$ mal
mal $\rightarrow$ cit
